# Supplementary material for: Disruption of the SYNGAP1 PDZ ligand motif accelerates differentiation of human iPSC-derived GABAergic neurons
Source: bioRxiv. 2026 Feb 25:2026.02.24.707848. Preprint. [Version 1] doi: 10.64898/2026.02.24.707848 (PMC13160062; doi:10.64898/2026.02.24.707848)
Supplement: Supplement 1 — Supplementary Figure 1 A. Generation of SYNGAP1 PDZ ligand mutant cell line. (A) Scheme of SYNGAP1 modular structure with PH, C2, RASGAP, DUF3498 (domain of unidentified function 3498) protein domains, together with the PDZ ligand at the c-terminal region QTRV. The cartoon shows the replacement of c-terminal sequence QTRV, by QIRE. B. Strategy of the two amino acids substitution and sequence confirmation for the double point mutation of SYNGAP1 PDZ region on the WT (03231) genetic background. iPSC generation showing pluripotent stem cells markers OCT4 and SSE4. C. Quantitation of SYNGAP1 total levels by MS in NPCs shows no changes in SYNGAP1 total protein levels in SYNGAP1 PDZ mutant compared to its isogenic control, using the SYNGAP1 peptide 493AIEEMRLIGQK504 for targeted MS analysis. [file media-1.pdf]

Suppl. Fig1

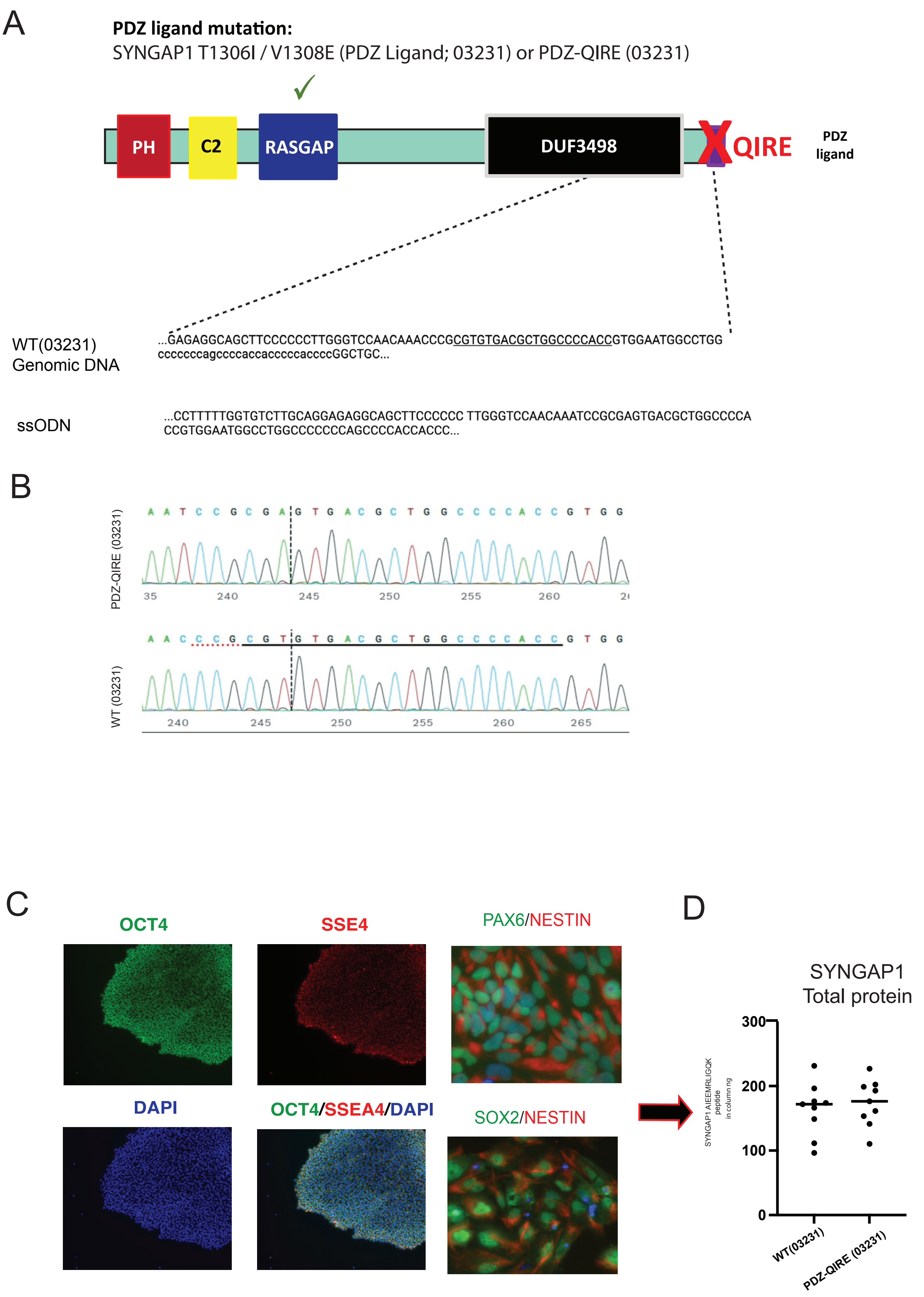
A
PDZ ligand mutation:
SYNGAP1 T1306I / V1308E (PDZ Ligand; 03231) or PDZ-QIRE (03231)
PH
C2
RASGAP
DUF3498
QIRE
PDZ ligand
WT(03231) Genomic DNA
...GAGAGGCAGCTTCCCCCCTTGGGTCCAACAAACCCGCGTGTGACGCTGGCCCCACCGTGGAATGGCCTGG cccccccagccccaccaccccccaccccGGCTGC...
ssODN
...CCTTTTTGGTGTCTTGCAGGAGAGGCAGCTTCCCCCC TTGGGTCCAACAAATCCGCGAGTGACGCTGGCCCCA CCGTGGAATGGCCTGGCCCCCCCAGCCCCACCACCC...
B
PDZ-QIRE (03231)
A A T C C G C G A G T G A C G C T G G C C C C A C C G T G G
WT (03231)
A A C C C G C G T G T G A C G C T G G C C C C A C C G T G G
C
OCT4
SSE4
PAX6/NESTIN
DAPI
OCT4/SSEA4/DAPI
SOX2/NESTIN
D
SYNGAP1 Total protein
SYNGAP1 AIEEMRLIGQK peptide in column ng
300
200
100
0
WT(03231)
PDZ-QIRE (03231)
