# Supplementary figures and images for: Disruption of the SYNGAP1 PDZ ligand motif accelerates differentiation of human iPSC-derived GABAergic neurons
Source: bioRxiv. 2026 Feb 25:2026.02.24.707848. Preprint. [Version 1] doi: 10.64898/2026.02.24.707848 (PMC13160062; doi:10.64898/2026.02.24.707848)

Phosphopeptides disregulated in SYNGAP1 PDZ-QIRE(03231) GABAergic iN

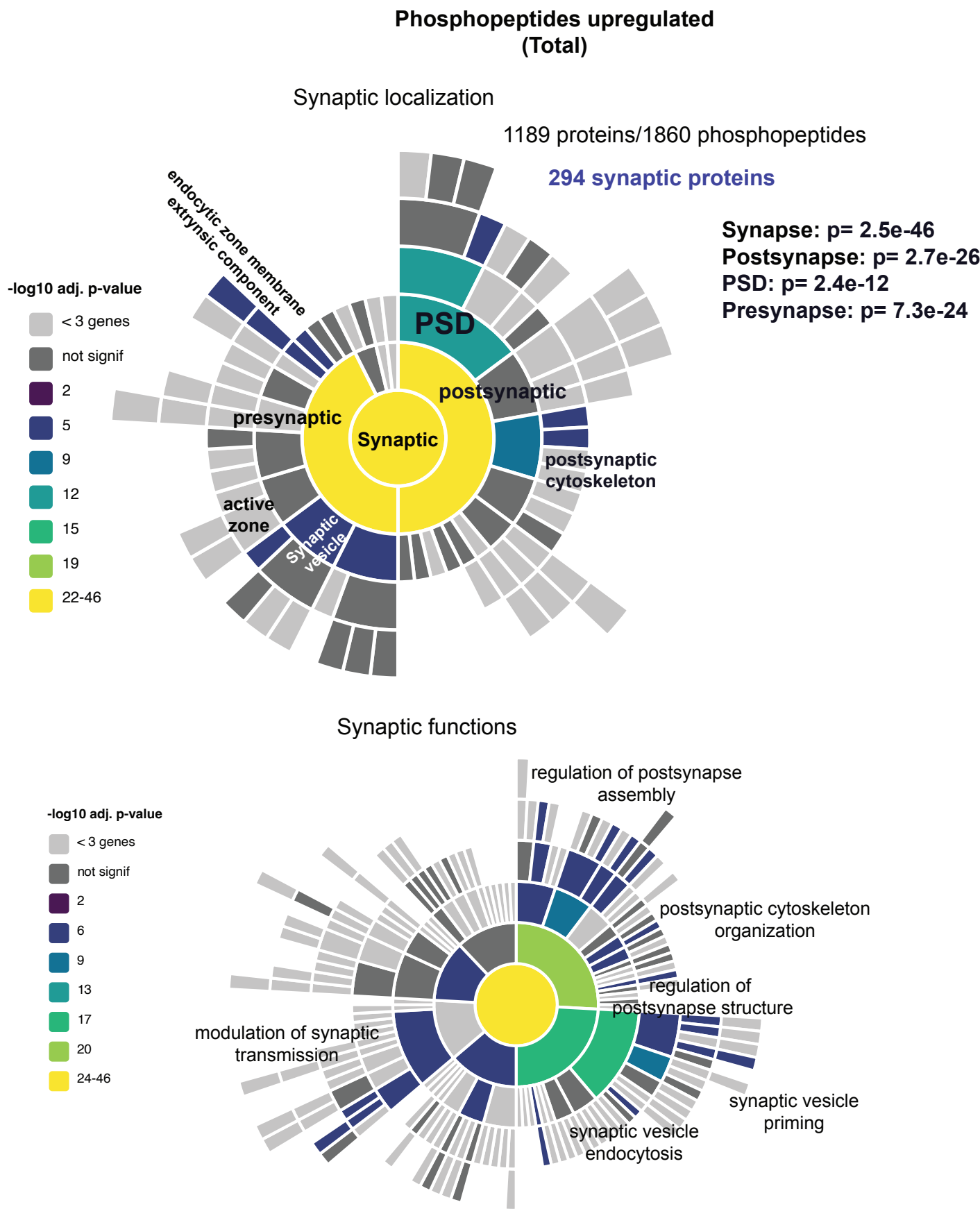

Supplement: Supplement 2 — Supplementary Figure 2 Figure shows SynGO analysis (left) of total number of phosphopeptides upregulated in PDZ-QIRE (03231) GABAergic iN compared to its isogenic control WT (03231). Sun plot shows an enrichment of presynaptic and postsynaptic proteins corresponding to structural, cytoskeletal, membrane components of the PSD together with presynaptic membrane proteins, synaptic vesicle proteins and components of the active zone. Right plots show analysis of synaptic functions enriched within upregulated proteins including organization of the pre and post signaling machinery, assembly of the synapse and synaptic vesicle release. Lower plots show upregulated phosphorylation sites that corresponds only to upregulated proteins. The analysis shows that a large proportion of the observed changes in protein phosphorylation match upregulation in protein levels. [file media-2.pdf]
