# Supplementary material for: Disruption of the SYNGAP1 PDZ ligand motif accelerates differentiation of human iPSC-derived GABAergic neurons
Source: bioRxiv. 2026 Feb 25:2026.02.24.707848. Preprint. [Version 1] doi: 10.64898/2026.02.24.707848 (PMC13160062; doi:10.64898/2026.02.24.707848)
Supplement: Supplement 3 — Supplementary Figure 3 A. Analysis of changes in protein phosphorylation curated against changes in protein levels. A total of 1088 phosphopeptides occurred in 797 proteins that were not found to be upregulated in the SYNGAP1 PDZ-QIRE (03231) genotype. A total of 117 of these proteins were identified as synaptic proteins by SynGO analysis. These proteins were enriched in synaptic and postsynaptic components (Sun plot/SynGO), but not presynaptic proteins. Colored circles show different functional groups within the synapse, including components of the SYNGAP1 interactome such as ANKS1B, ANK3, MAPK1, CYFIP1, together with GTP signaling proteins (green), structural and signaling components of the PSD (yellow), presynaptic proteins (blue), RNA binding/ribosomal proteins (purple) protein kinases (pink) and channels (dark blue). B. Protein phosphorylation sites upregulated in non-synaptic components were enriched in RNA processing, Nucleotide excision repair development, gene expression, cellular growth, and RNA PTMs. [file media-3.pdf]

Suppl. Figure 3

A

Phosphopeptides upregulated (no changes in total protein levels)

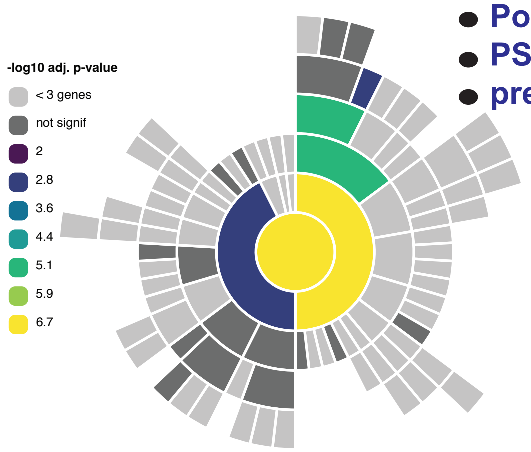

797 protein/1090 phosphorylation sites
129 synaptic proteins

- **Synapse: p=1.13e-7**
- **Postsynapse: p=1.6e-7**
- **PSD: p=9.25e-6**
- **presynapse p=0.003**

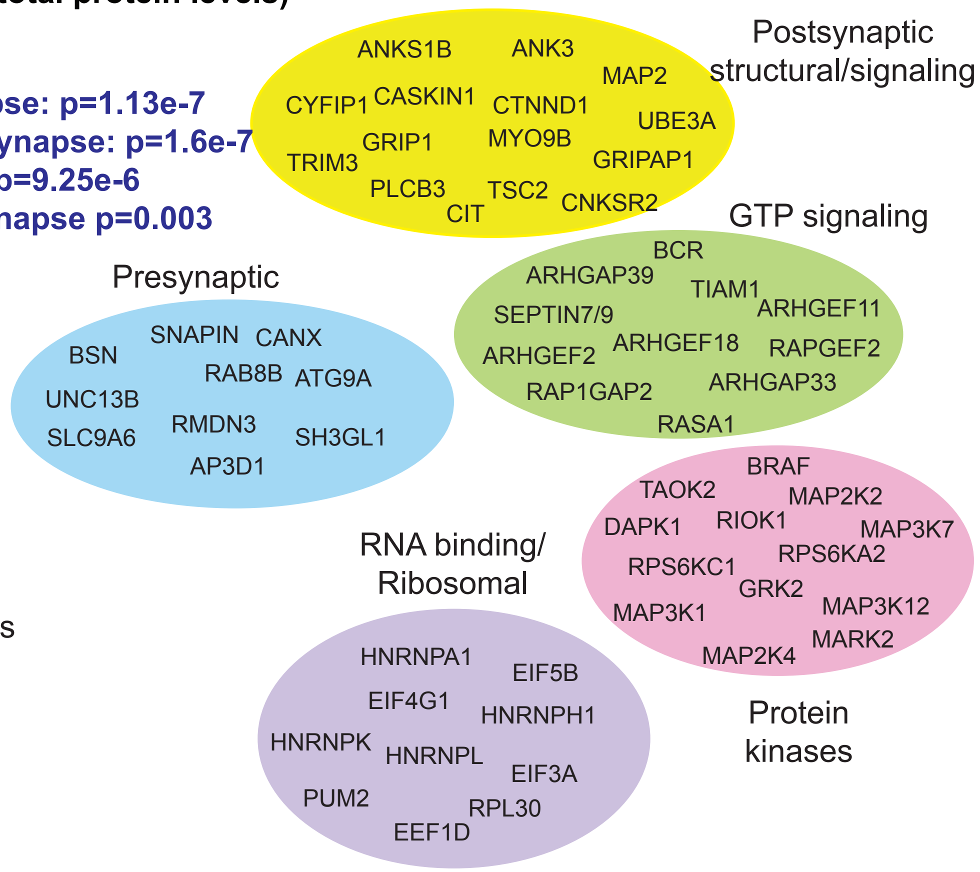

B

Disregulated phosphopeptides: (Non-synaptic component)

Top canonical pathways

Name	p-value
Processing of Capped Intron-Containing Pre-mRNA	7.08E-21
RNA Polymerase II Transcription	3.16E-11
NER (Nucleotide Excision Repair, Enhanced Pathway)	4.80E-09
Nucleotide Excision Repair	9.27E-08
Mitotic Metaphase and Anaphase	9.34E-08

Molecular and Cellular Functions

Name	p-value range	# Molecules
RNA Post-Transcriptional Modification	2.09E-03 - 2.60E-31	65
Gene Expression	4.00E-05 - 7.38E-22	166
Cellular Function and Maintenance	2.44E-03 - 1.28E-20	280
Cellular Development	2.10E-03 - 1.44E-19	233
Cellular Growth and Proliferation	2.35E-03 - 1.44E-19	232
